# Bailout transcatheter mitral valve implantation for extensive valvular and atrial calcifications during triple-valve surgery in a patient with long-lasting renal replacement therapy: a case report

**DOI:** 10.1093/ehjcr/ytag239

**Published:** 2026-03-31

**Authors:** Giuseppe Verolino, Kim E H M van der Velden, Barthel W Sauren, Suzanne Kats, Roberto Lorusso

**Affiliations:** Invasive Cardiology, IRCCS Istituto Auxologico Italiano - San Luca Hospital, Piazzale Brescia 20, 20148 Milan, Italy; Department of Cardiothoracic Surgery Heart+Vascular Centre, Maastricht UMC+, P. Debyelaan 25, 6669 Maastricht, The Netherlands; Cardiovascular Research Institute Maastricht (CARIM), Universiteitssingel 50, 632, 6229 ER Maastricht, The Netherlands; Department of Anesthesiology and Pain Medicine, Maastricht UMC+, P. Debyelaan 25, 6669 Maastricht, The Netherlands; Department of Anesthesiology and Pain Medicine, Maastricht UMC+, P. Debyelaan 25, 6669 Maastricht, The Netherlands; Department of Cardiothoracic Surgery Heart+Vascular Centre, Maastricht UMC+, P. Debyelaan 25, 6669 Maastricht, The Netherlands; Department of Cardiothoracic Surgery Heart+Vascular Centre, Maastricht UMC+, P. Debyelaan 25, 6669 Maastricht, The Netherlands; Cardiovascular Research Institute Maastricht (CARIM), Universiteitssingel 50, 632, 6229 ER Maastricht, The Netherlands

**Keywords:** End-stage renal disease, Calcified amorphous tumour, Triple valve surgery, Minimally invasive procedures, Open surgery transcatheter heart valve implantation, Case report

## Abstract

**Background:**

We present a successful case of triple-valve surgery, including bailout open-heart transcatheter mitral valve implantation, in a haemodialysis patient with extensive intracardiac calcifications.

**Case summary:**

A middle-aged woman with exercise-induced dyspnoea and an end-stage kidney disease on renal replacement therapy was admitted to the emergency department for severe hypotension and bradycardia. A high-degree AV block was diagnosed. Multimodal imaging exams also revealed a triple severe valvular disease (mitral stenosis, aortic stenosis, and tricuspid regurgitation) with an unclear cardiac mass enclosed within the left atrial wall. To avoid potentially dangerous decalcification for valve replacement, a combined traditional surgical approach and transcatheter valve implantation have been successfully applied.

**Discussion:**

The current case confirms that open-heart transcatheter valve implantation at the mitral position in the presence of marked calcifications involving the valve apparatus, atrial, and ventricular walls is a valuable option, potentially avoiding dangerous decalcification, achieving effective mitral valve stenosis relief, and shortening surgical times.

Learning points

- Hybrid valve implantation procedures might represent a successful and effective bailout intervention, particularly in combined valve surgery with specific conditions.
- In the presence of massive valve and extra-valvular calcification, considering transcatheter valve implantation during open surgery could prevent perioperative cardiac-related structure rupture. allowing a decalcification-free operation.

## Introduction

Patients with valvular heart disease and end-stage renal disease (ESRD) face a heightened surgical risk due to the frequent occurrence of combined valvulopathies and the potential for prosthesis damage caused by calcific degeneration.^1^ Additionally, robust and comprehensive data regarding this specific patient population remain scarce in the available literature.

## Summary figure

**Figure:**
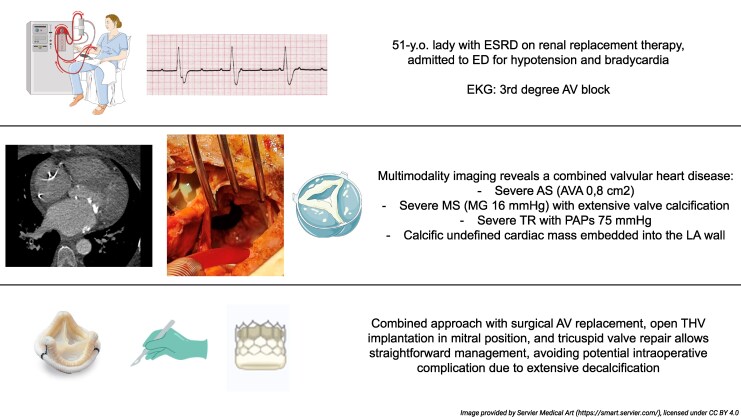
Patient’s medical course, from clinical presentation in ED (upper panel), valvular heart disease diagnosis (middle panel), to surgical management (bottom panel).

## Case presentation

A 51-year-old woman presented with recurrent episodes of dizziness, light-headedness, and exertional dyspnoea with thoracic pain radiating to the upper back. She was asymptomatic at rest. On admission to the emergency room, she exhibited hypotension (77/50 mmHg) and marked bradycardia (40 bpm). Physical examination revealed pallor and nausea in the absence of clinical signs or symptoms of acute infectious or respiratory disease. Cardiac auscultation identified a systolic murmur at the second right parasternal intercostal space and along the 4th/5th left intercostal midclavicular space. No peripheral oedema was noted. The patient had been undergoing intermittent haemodialysis for eight years due to drug-induced nephropathy. Her medical history was otherwise unremarkable for thromboembolic, haematologic, malignant, or inflammatory disorders. The electrocardiogram (EKG) revealed a third-degree atrioventricular block. Transthoracic (TTE) and transoesophageal echocardiography (TEE) showed preserved left and right ventricular function, but a combined valve pathology was present:

- Severe aortic valve (AV) stenosis (AVA decreased from 1.2 cm^2^ to 0.9 cm^2^ over a few months) with mild regurgitation and severe concentric left ventricular hypertrophy.
- Severe mitral valve stenosis (mean gradient 16.6 mmHg, MVA 1.1 cm) with mild regurgitation and severe left atrial dilatation, accompanied by extensive atrial and valvular/subvalvular calcifications.
- Severe tricuspid regurgitation resulting in severe pulmonary hypertension (right ventricular systolic pressures of 70–75 mmHg) and moderate-to-severe right atrial dilatation.

**Figure 1:**
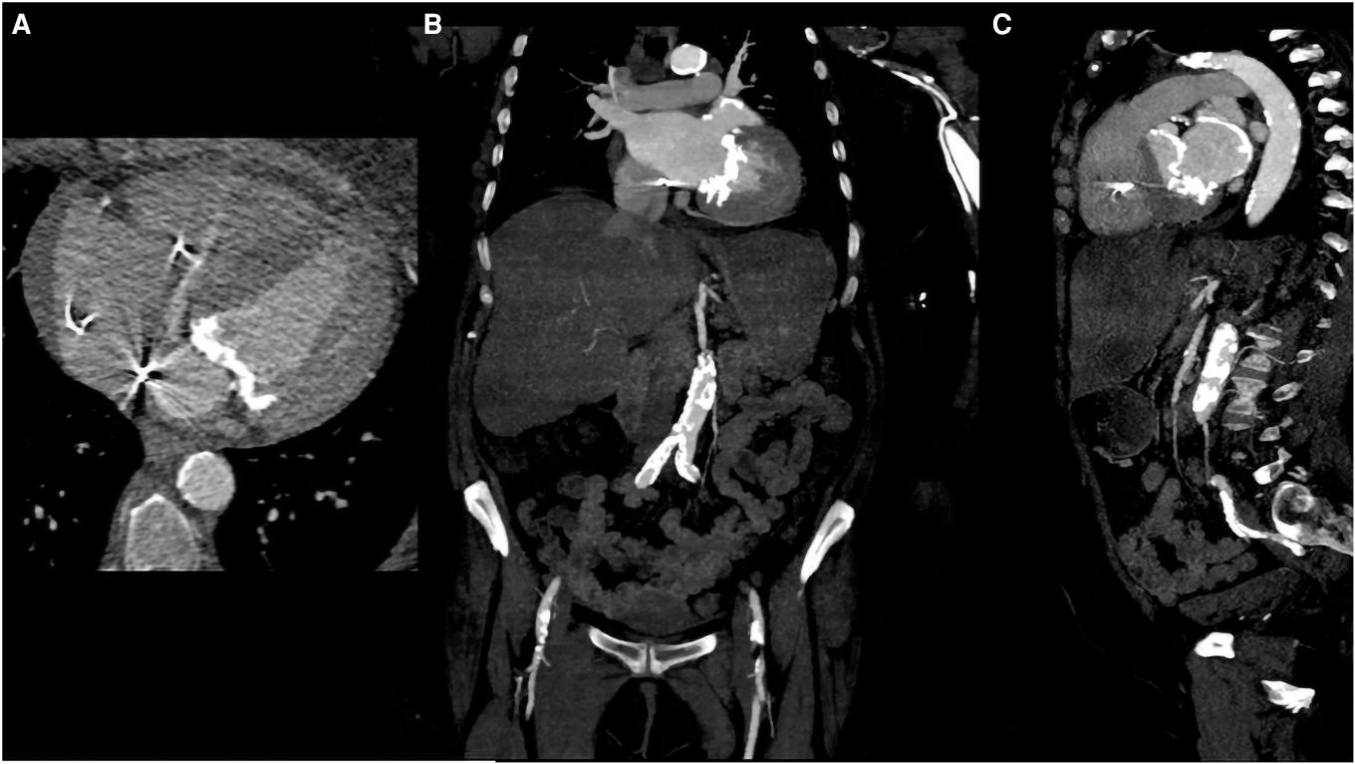
This cardiac CT image shows explicit calcifications within the heart. Main sites of calcifications are located at the site of the mitral annulus and involve a part of the nearby atrial wall. Panel *A*, axial view showing wide mitral calcification; panel *B*, coronal view; panel *C*, sagittal view, displaying extensive calcifications within left atrium, left ventricle, and mitral valve apparatus.

The coronary angiogram revealed no significant coronary stenosis. However, cardiac computed tomography (CCT) confirmed massive calcifications involving both the mitral and aortic valves. Such calcifications extended to the mitro-aortic continuity and the posterior ventricular free wall, as well as the presence of a thrombus in the left atrium (*Figure 1*, panel A-B). Additionally, massive calcification of the left atrial site was observed, originating from the mitral annulus and extending towards the pulmonary veins (*Figure 1*, panel C). Laboratory findings confirmed severe renal failure, with no clinical or biochemical evidence of systemic inflammation or infection.

## Management

**Figure 2:**
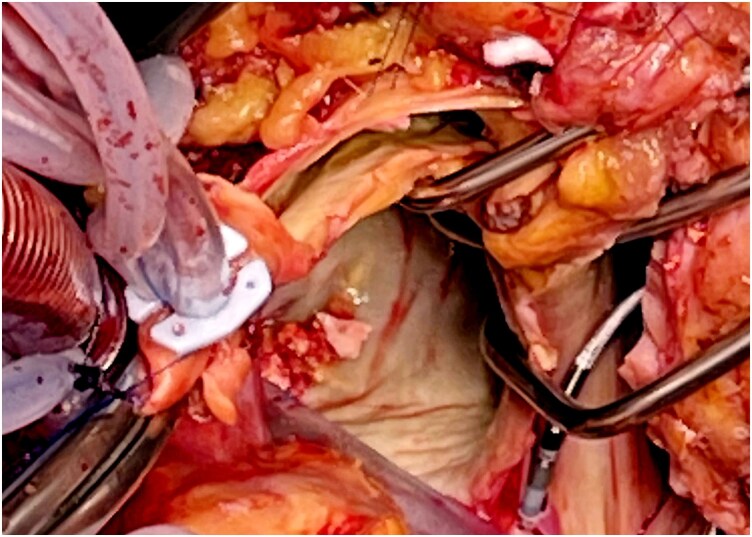
This picture shows calcified fragments after surgical opening of the left atrium. These calcifications, extending towards the mitral valve, were later histologically identified as a cardiac amorphous tumor.

**Figure 3:**
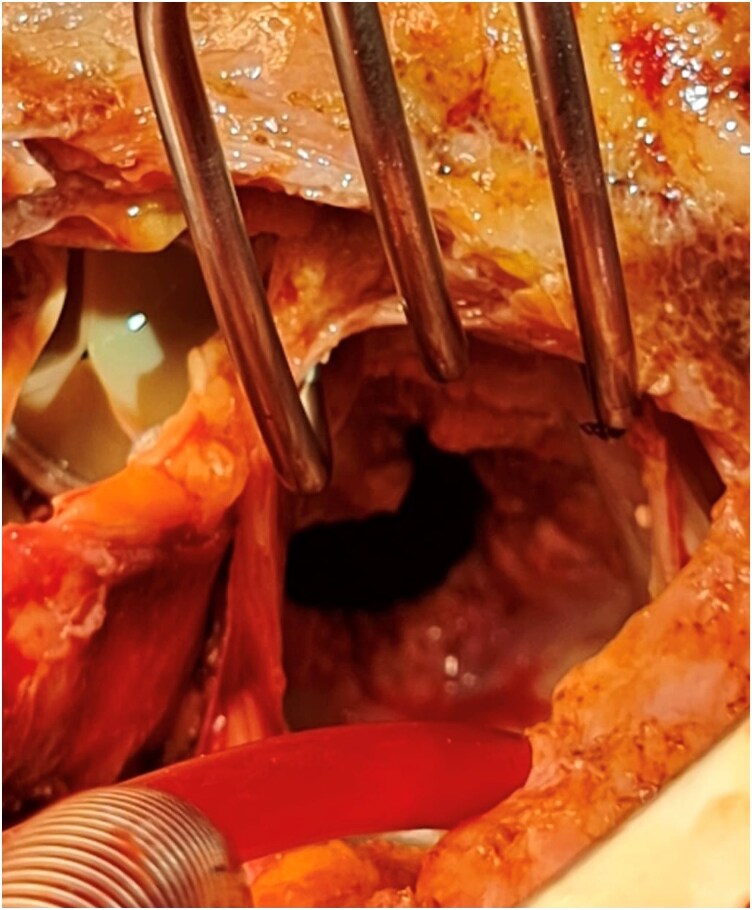
This picture shows the bailout, direct, open-heart transcatheter mitral valve implantation. For this procedure, an Edwards Sapien valve size 26 was used.

**Figure 4:**
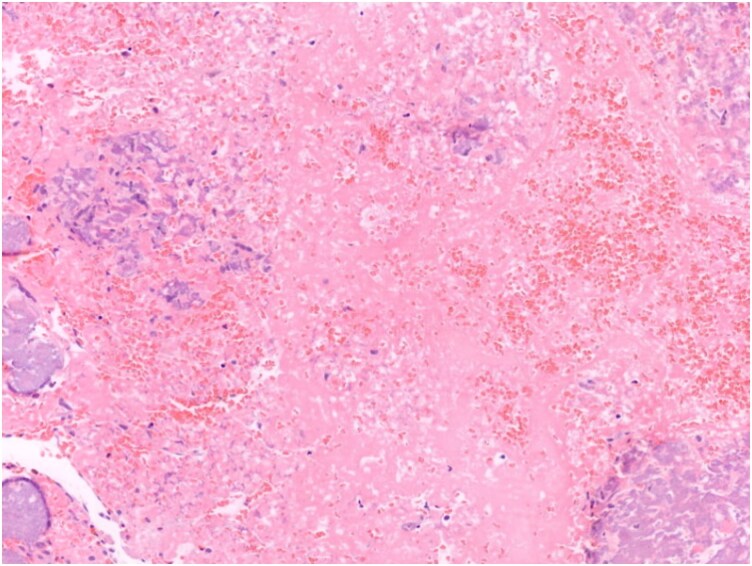
Histologic examination showing fragments with dystrophic calcifications and moderate inflammation. This was consistent with the diagnosis of calcified amorphous tumour.

Primarily, the patient underwent pacemaker implantation. The patient was subsequently scheduled for combined aortic and mitral valve replacement, along with concomitant tricuspid valvuloplasty. Intraoperative TEE revealed a large mobile structure in the left atrium (see Supplementary material online, *Video S1*–S2). Despite challenging native AV resection and decalcification, an extensive mitral valvular and subvalvular calcification was observed through the AV view. AV replacement was performed using a biological prosthesis. Following biatrial exposure (via a Guiraudon incision) to optimize visualization of the left atrium and mitral valve, massive calcification involving the entire mitral valve apparatus and the adjacent left atrial wall was identified. In detail, a large spot of the posterior atrial wall, encompassing three irregular beige-brown tissue fragments with partial calcifications (diameters of 5, 10, and 11 millimetres, respectively), was identified and easily resected (*Figure 2*). No signs of infective endocarditis were detected, as confirmed by histological examination. The mitral valve calcifications deeply affected the valvular apparatus, the adjacent left atrial wall, and the posterior left ventricular wall. Considering the high risk of atrioventricular groove or free-wall rupture during or after annular decalcification, the decision was made to directly implant an aortic THV (Sapien 3, size 26 mm) in the mitral position (*Figure 3*). A water test demonstrated appropriate valve competence without paravalvular leakage. Tricuspid valvuloplasty was performed using the DeVega technique on the beating heart after declamping and deairing of the left-sided cardiac chambers. Histological examination revealed fragments of acellular, amorphous calcified material (*Figure 4*). The postoperative course was uneventful, and post-operative TEE confirmed the good procedural outcome (see Supplementary material online, Videos). At the 30-day follow-up, the patient was alive and asymptomatic.

## Discussion

Calcified amorphous tumour (CAT), first described by Reynolds and colleagues in 1997 in a case series on dialysis patients, remains poorly understood in terms of its pathogenesis.^2^ Various etiologist have been suggested so far, including hypercoagulability disorders (e.g. antiphospholipid syndrome), disturbances of calcium-phosphate metabolism (e.g. hyperparathyroidism), haemodialysis, and chronic inflammation.^3–5^ In the present case, cardiac computed tomography (CCT) was performed as part of the preoperative assessment for triple-valve surgery. The presumed thrombus in the left atrium, detected on CCT, was an incidental finding. Although CCT is not typically considered the first-line modality for the assessment of cardiac masses, it proved valuable as a complementary tool in transthoracic echocardiography (TTE). Advances in cardiac magnetic resonance imaging (CMR) and improvements in the quality of TTE and transoesophageal echocardiography (TEE) have substantially enhanced diagnostic accuracy. Despite the high effectiveness of both CMR, TTE, and TEE in distinguishing benign from malignant masses, histopathological examination remains the gold standard.^6,7^

The question arises whether cardiac masses that appear benign on CMR (including cardiac CATs) should be surgically removed in asymptomatic patients or those with a high or extremely high surgical risk. Surgical excision is often considered because of the potential risk of systemic embolization.^8^ In our case, the indication for surgical intervention was clear owing to severe multi-valvular disease. Although previous case reports and case series described the association between intracardiac calcification and end-stage renal disease (ESRD), the distribution of calcification in this case was unusually extensive, not exclusively including the known preferential sites of the mitral annulus and mitral leaflets but also the aortic valve and the walls of the left atrium and ventricle,^9^ as illustrated in *Figure 2*. The extent of the intracardiac calcifications in this patient may be attributed to long-term haemodialysis.

Management of mitral stenosis with mitral annular calcification (MAC) is described in two registries from Praz and Russell, reporting successful transcatheter mitral valve implantation (THV) in 13 and 6 patients, respectively.^10,11^ In both cases, the Sapien 3 prosthesis was the preferred choice, as in our case. However, these series did not include cases of combined valvular heart defects. In line with our case, Gollmann-Tepeköylü and Ahmad describe successful hybrid surgical procedures in patients with significant mitral annulus calcification, without damage to surrounding structures or left ventricular outflow tract (LVOT) obstruction.^12,13^

A hybrid approach, which combines a conventional aortic valve replacement with a tissue valve and a direct open-heart transcatheter mitral valve implantation, offers a safe and successful treatment option in potentially life-threatening situations. Extensive or even partial decalcification of the mitral valve annulus or left atrial wall can carry a significant risk of intra-operative or perioperative atrio-ventricular groove or ventricular free-wall rupture, requiring a time-consuming and complex management. To the best of our knowledge, our case describes the first triple-valve surgery performed for extensive calcification in combined valvular disease (AVR, THV for MAC, and tricuspid repair). In such challenging scenarios, isolated or combined open-heart THV and traditional surgery approaches should be considered.^14^

## Conclusion

A hybrid surgical approach combining conventional valve surgery with open-heart transcatheter valve implantation may represent an effective strategy to address extremely high-risk situations.^15^ CAT is a rare, benign neoplasm of the heart that is frequently associated with haemodialysis and ESRD. Depending on its location, cardiac CATs pose a high risk of intra- or perioperative complications due to extensive calcifications. The combination of diagnostic modalities, including cardiac CAT, cardiac magnetic resonance imaging (CMR), and echocardiography, can lead to an accurate diagnosis.

## Lead author biography


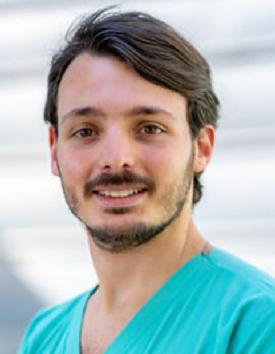


Dr. Verolino serves as an interventional cardiologist at IRCCS Istituto Auxologico Italiano - San Luca Hospital in Milan (Italy); moreover, he is currently a Ph.D. Fellow at the Cardiovascular Research Institute (CARIM) - Maastricht University (The Netherlands). His research interests are focused on novel insights into valvular heart disease treatment, coronary artery disease (acute and chronic coronary syndromes), and structural heart disease, such as patent foramen ovale.

## Supplementary Material

ytag239_Supplementary_Data

## Data Availability

All data are incorporated into the article and its online Supplementary material.

## Notes

**Consent:** The authors confirm that a written consent for the submission and publication of this case report, including the images and associated text, has been obtained from the patient in line with COPE guidelines.
